# Moxibustion improves hypothalamus *Aqp4* polarization in APP/PS1 mice: Evidence from spatial transcriptomics

**DOI:** 10.3389/fnagi.2023.1069155

**Published:** 2023-02-02

**Authors:** Shuqing Liu, Hongying Li, Yuan Shen, Weikang Zhu, Yong Wang, Junmeng Wang, Ning Zhang, Chenyu Li, Lushuang Xie, Qiaofeng Wu

**Affiliations:** ^1^Acupuncture and Moxibustion School, Chengdu University of Traditional Chinese Medicine, Chengdu, Sichuan, China; ^2^National Center for Mathematics and Interdisciplinary Sciences, CEMS, NCMIS, MDIS, Academy of Mathematics and Systems Science, Chinese Academy of Sciences, Beijing, China; ^3^College of Basic Medicine, Chengdu University of Traditional Chinese Medicine, Chengdu, Sichuan, China

**Keywords:** moxibustion, Alzheimer’s disease, spatial transcriptomics, aquaporin-4, mitochondrial respiratory chain, hypothalamus

## Abstract

Aquaporin-4 (AQP4) is highly polarized to perivascular astrocytic endfeet. Loss of AQP4 polarization is associated with many diseases. In Alzheimer’s disease (AD), AQP4 loses its normal location and thus reduces the clearance of amyloid-β plaques and tau protein. Clinical and experimental studies showed that moxibustion can improve the learning and memory abilities of AD. To explore whether moxibustion can affect the polarization of AQP4 around the blood-brain barrier (BBB), we used spatial transcriptomics (ST) to analyze the expression and polarization of *Aqp4* in wild-type mice, APP/PS1 mice, and APP/PS1 mice intervened by moxibustion. The results showed that moxibustion improved the loss of abnormal polarization of AQP4 in APP/PS1 mice, especially in the hypothalamic BBB. Besides, the other 31 genes with *Aqp4* as the core have similar depolarization in APP/PS1 mice, most of which are also membrane proteins. The majority of them have been reversed by moxibustion. At the same time, we employed the cerebrospinal fluid circulation gene set, which was found to be at a higher level in the group of APP/PS1 mice with moxibustion treatment. Finally, to further explore its mechanism, we analyzed the mitochondrial respiratory chain complex enzymes closely related to energy metabolism and found that moxibustion can significantly increase the expression of mitochondrial respiratory chain enzymes such as Cox6a2 in the hypothalamus, which could provide energy for mRNA transport. Our research shows that increasing the polarization of hypothalamic *Aqp4* through mitochondrial energy supply may be an important target for moxibustion to improve cognitive impairment in APP/PS1 mice.

## Introduction

Alzheimer’s disease (AD) is an irreversible neurodegenerative disease characterized by progressive memory loss, cognitive impairment, and emotional disorder (Scheltens et al., 2021). Unfortunately, currently available medications for AD may only temporarily and modestly improve cognitive symptoms. Thus, great efforts have been made to explore the mechanisms of AD. In the most recent times, the dysfunction of the blood-brain barrier (BBB), a tightly regulated barrier in the central nervous system, has been considered an important target involved in AD progression, eliciting a peripheral immune and inflammatory response (Huang et al., 2020).

Aquaporin-4 (AQP4), a kind of aquaporin channel, is highly expressed in astrocytes and mediates water exchange across the BBB (Salman et al., 2022). Perivascular enrichment of AQP4 at the BBB suggests a role in glymphatic function. It serves as a clearance pathway for protein species such as amyloid-β and tau, which accumulate in the brain in AD (Ding et al., 2021). The predominant subcellular localization of AQP4 is in the astrocyte endfeet membranes, directly in contact with the brain capillaries, with a low but significant concentration in non-endfeet membranes, such as those astrocyte membranes (Salman et al., 2022). Recently, it was demonstrated that AQP4 localization is dynamically regulated at the subcellular level, affecting membrane water permeability. Aging and other CNS disruptions are associated with the polarized expression of AQP4 in the astrocyte endfeet membranes in the BBB (Hoshi et al., 2012).

Moxibustion and acupuncture are important means of treating AD in traditional Chinese medicine (Cao et al., 2016; Rigun et al., 2021). Although the beneficial effects of moxibustion and acupuncture for AD have been primarily studied both preclinically and clinically (Zhou et al., 2014; Xie et al., 2021; Huang et al., 2022; Lai et al., 2022; Zhang et al., 2022), the real efficacy of these treatments on AD remains inconclusive, and the underlying mechanisms are largely unexplored. It has been shown that moxibustion can increase the flow velocity of cerebrospinal fluid and increase the expression of AQP4 in the cortex of mice (Chen et al., 2021), but it is uncertain whether moxibustion can induce the polarity or dynamical localization of AQP4. Meanwhile, the potential mechanism is unknown yet.

In this study, spatial transcriptomics (ST) technology was used, and the marker genes of the astrocyte and endotheliocyte were used to select the perivascular astrocytes and BBB complex, by which we can obtain the distribution characteristics of the *Aqp4* gene in each brain region. Then, a formula for the *Aqp4* ratio was introduced, which calculated the proportion of *Aqp4* mRNA in the astrocyte endfoot to that in the whole astrocyte. We used the formula result to evaluate the polarization of *Aqp4* in each brain region and finally analyze other polarization genes related to *Aqp4* and mitochondrial respiratory chain-related genes. In this approach, we aimed to study the mechanism of moxibustion promoting AQP4 polarization in AD from the perspective of transcriptomics.

## Materials and methods

### Animals

All experimental procedures were approved by the Animal Care and Use Committee of Chengdu University of Traditional Chinese Medicine (ethics number: 2018–10). All experimental mice were purchased from Cavens Biogle (Suzhou) Model Animal Research Co., Ltd. [license number: SCXK (su) 2018–0002], were 5 months old, and weighed 28.0 ± 2 g. The WT mice were male C57BL/6J mice, while the AD model mice were male APP/PS1 double transgenic mice. Mice were housed in a vivarium environment under a 12-h light-dark cycle (temperature 20 ± 2°C, humidity 50–65%) and had free access to diet and drinking water. All the mice were screened by the Morris water maze test after a week of adaptation. Then, APP/PS1 mice were randomly divided into the AD model group (APP/PS1) and the moxibustion group (APP/PS1 + MOX), and C57BL/6J wild-type mice were used as the control group (WT group).

### Morris water maze (MWM) test

**FIGURE 1:**
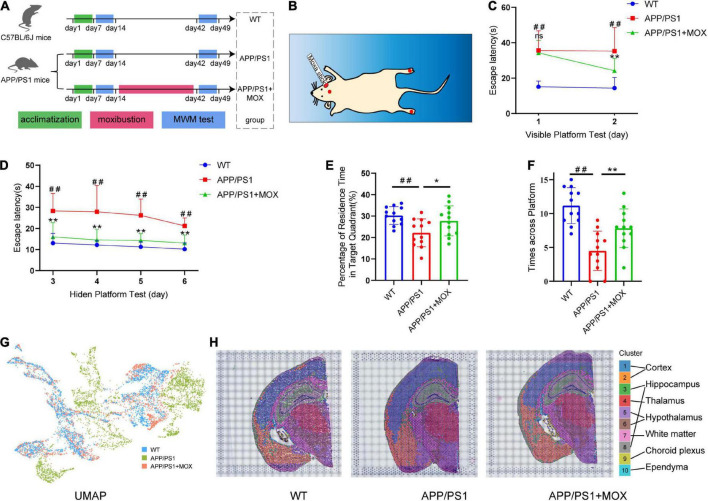
Moxibustion rescued defects in memory and spatial transcriptomic (ST) characteristic distribution in APP/PS1 mice. **(A)** Flowchart of the experiment. The Morris water maze (MWM) test was carried out on 5-month-old mice. After 7 days of the MWM test, the mice were treated for 4 weeks continuously, and then the MWM test was conducted again. **(B)** Schematic of the locations of the acupoints in mice. The red dot on the head of the mouse is the GV20 acupoint, and the foot center is the KI1 acupoint **(C,D)**. Learning curves of wild-type (WT), APP/PS1, and APP/PS1 + MOX mice in the MWE test with visible **(C)** and hidden **(D)** platforms. **(E,F)** Percentage of residence time in the target quadrant **(E)** and crossing platform times **(F)** of mice. **(G)** UMAP plot shows the differences among groups. There was more overlap between APP/PS1 + MOX (red) and WT (blue), but APP/PS1 (green) is relatively separate. **(H)** Cluster graph in each sample obtained by Graph-based clustering. Different colors and lines corresponded to different clusters and brain regions. One-way ANOVA test with post-test ^#^*P* < 0.05, ^##^*P* < 0.01, APP/PS1 vs. WT; *P < 0.05, **P < 0.01, APP/PS1 + MOX vs. APP/PS1; ns. *P* > 0.05.

The MWM test was conducted in a circular tank (diameter, 90 cm; height, 30 cm) in a dimly lit room. The water temperature was kept at 22–25°C to prevent the mice from floating. A removable circular platform (diameter, 9.5 cm; height, 28 cm) was equipped 1.5 cm below the milky water surface in one of the quadrants. The pool area was divided into four equal quadrants, and markers were added in different shapes by affixing color cards. During the 4 days of visible platform training, each mouse received 4 training trials every day. Then, during the 4 consecutive days of hidden platform training, mice were trained for four trials each day. After the last trial of training, all mice were given one probe trial for 90 s of searching (probe trial) without a platform. The time spent by the mice in each quadrant was measured; in the meantime, total swimming distance and crossing times in each quadrant were automatically recorded by the water maze system (Chengdu Techman Software Co., Inc.). Figure 1A shows the experimental flowchart.

### Moxibustion treatment

Treatments were performed after the MWM test. Acupoints “Yongquan” (KI1) and “Baihui” (GV20) were selected for intervention. The locations of the two acupoints were determined according to the Government Channel and Points Standard GB12346-90 of China and “The Veterinary Acupuncture of China” (Figure 1B). In the APP/PS1 + MOX group, mice were treated with a moxa stick (diameter, 0.5 cm; length, 18 cm), which was ignited and hung 1.5 cm above acupoints. The mice in the WT and APP/PS1 groups were only fastened without moxibustion. Our previous studies (Huang et al., 2022; Zhang et al., 2022) proved that the treatment was effective when given for 30 min/day, 5 consecutive days per week, for 4 weeks.

### Sample collection

After 5 days of treatment and the MWM test, all mice were anesthetized with 1% pentobarbital sodium (3 mL/kg). For ST, whole brains were isolated using an aseptic technique, embedded in OCT, and snap-frozen quickly. For immunofluorescence (IF), tissues were fixed in formaldehyde and embedded in paraffin. For transmission electron microscopy (TEM), fresh brains were placed on the icebox and the hippocampus was quickly stripped off. Then, the tissues were cut to 2 mm^3^, and the tissues were moved to the EP tube filled with fresh TEM fixatives for further fixation. For RT-qPCR, hypothalamus tissues were separated and kept frozen at −80°C.

### Spatial transcriptomics (ST)

#### Sample preparation for spatial transcriptomics

The brains were coronally cryosectioned at a thickness of 10 μm using Cryostat Microtome (Leica CM3050, Germany). Then, 5–10 sections were selected to be stained with HE, photographed, and tested for RNA integrity number (RIN) value.

#### Spatial transcriptomics library preparation, sequencing, and data preprocessing

The ST libraries were prepared using the 10 × Genomics Chromium system. Sample permeabilization was referred to as Visium Spatial Tissue Optimization Kit (1000184, 10x Genomics, USA). After tissue permeabilization and cDNA synthesis, library preparation, and high-throughput sequencing, a total of 11,850 spots and about 1.7 × 10^9^ reads were targeted for capture from four mouse samples. The raw data were processed using the analysis pipeline Space Ranger (1.0.0) for sample demultiplexing, barcode processing, read alignment to the mouse reference genome (mm10), and gene counting.

#### Spatial transcriptomic data analysis

After quality control in Seurat packages (version 4.1.0), sample integration was done using Space Ranger. Cell clustering, tSNE analysis, gene distribution analysis, and selection of a subset were realized through Loupe Browser. The results of differentially expressed genes (DEGs) were obtained by selecting the data after merging and SCTransform through Seurat packages and having differential gene expression analysis through the edgeR package (version 3.26.8). The pathway and process enrichment analyses were carried out using Metascape (Zhou et al., 2019). The GO term scores were calculated using the AddModuleScore function in Seurat.

#### Screening methods for astrocytes adjacent to blood vessels

Perivascular astrocytes (AC) were selected as the intersection of spots with glial fibrillary acidic protein (*Gfap*) gene expression > 0 and *Aqp4* expression > 0. The spots in the glia limitans region, ependymal region, and choroid plexus region were eliminated from the AC. BBB was selected as the intersection of spots with *Pecam1* expression > 0 and *Flt1* expression > 0. In addition, the spots in the choroid plexus region were eliminated from the BBB. The above filtering method was implemented by the Loupe Browser software. Then, the spatial information of the spots was used to filter out the adjacent AC and BBB for further analysis.

#### Methods for screening AC differentially expressed genes

The DEGs were screened with | Fold change| > 1.5 and a *p*-value adjustment of < 0.05.

#### Screening of the AD-related AEGs

A total of 204 AC marker genes were screened from the VascularSingleCells (Vanlandewijck et al., 2018) with fold change > 100 than other BBB cells. Among them, the genes that were endfoot-positive in more than 60% of astrocytes were called astrocyte endfoot genes (AEGs), and the genes with a gene ratio change of more than 0.15-fold in the APP/PS1 group were called AD-related AEGs.

#### Calculation of gene ratio

The gene ratio in AC was the amount of gene expression in adjacent BBB divided by the sum of gene expression in AC and BBB. The AC with multiple gene ratios took the maximum value.

### Acquisition and analysis of public RNA-seq data

**FIGURE 2:**
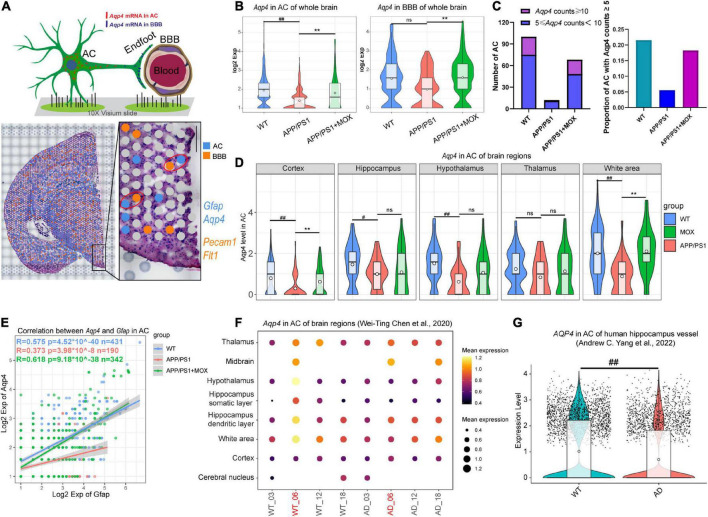
The reduction of *Aqp4* expression in perivascular astrocytes (AC) and the blood-brain barrier (BBB) in APP/PS1 mice. **(A)** Schematic diagram of screening methods for perivascular astrocytes. *Gfap* and *Aqp4* represent the signature genes of AC, while *Pecam1* and *Flt1* represent the signature genes of the BBB. **(B)** Violin plots of *Aqp4* expression in BBB and AC. **(C)** Barplots indicating the number and proportion of AC with high *Aqp4* expression in each group. **(D)** Violin plots of *Aqp4* expression in AC of different brain regions. **(E)** A scatter plot with a linear fit line showing the correlation between *Aqp4* expression and *Gfap* expression in AC of each group. R, Pearson’s correlation coefficient; p, Pearson’s correlation test *P*-value. **(F)** Bubble plot of *Aqp4* expression in AC of different age WT and AppNL-G-F mouse brain. **(G)** *Aqp4* expression in AC of the human hippocampus. One-way ANOVA test with post-test ^#^*P* < 0.05, ^##^*P* < 0.01, APP/PS1 vs. WT; ^**^P < 0.01, APP/PS1 + MOX vs. APP/PS1; ns. *P* > 0.05.

The public RNA-seq data in Figures 2F, G were acquired from the Gene Expression Omnibus (GEO) public database (GEO Accession Numbers: GSE152506 and GSE163577).^1^ The method of data preprocessing and nomenclature of cells or brain regions was the same as that described previously in this study (Chen et al., 2020; Yang et al., 2022). In addition, the selection of AC and the differential expression analysis were chosen in the same way as the ST analysis.

### Immunofluorescence (IF)

Brain tissues were sectioned into 10-μm-thick slices. Sectioned samples were deparaffinized in xylene, rehydrated in a series of graded alcohols, and subjected to antigen retrieval. Endogenous peroxidase was quenched with 3.0% hydrogen peroxide in methanol for 30 min. Sections were further blocked with 3.0% bovine serum albumin (BSA) in PBS, exposed to 0.5% Triton X-100 for 1 h to reduce non-specific antibody binding, and incubated with mouse AQP4 (ImmunoWay Biotechnology) or rabbit CD31 (Affinity Biosciences) antibodies (1:200) at 4°C overnight. Then, sections were washed with PBS three times and incubated with secondary antibodies (Alexa Fluor 488, cy3, Bioss, China, 1:200) for 2 h at 37°C. Photographs were taken using an immunofluorescent microscope (Leica, Germany) and analyzed for the relative%area positive expression or average optical density with the Leica Application Suite X (Leica, Germany) and ImageJ software.

### The proportion of AQP4-coated cerebral vessel analysis

After the IF test, six 0.2 mm × 0.2 mm regions were randomly selected from the cerebral cortex of each sample. The blood vessels were the vacuoles with CD31 expression, and the positive expression of AQP4 was the first 1% fluorescence intensity in the ImageJ software threshold. The cerebral vessels co-labeled with AQP4 were those that were surrounded by more than a quarter of AQP4-positive sites. Finally, the proportion of cerebral vessels co-labeled with AQP4 in this region was calculated.

### Transmission electron microscope (TEM)

After postfixation for 1 week, brain tissues were washed with PBS, postfixed with 1% OsO4 in PB at 4°C for 2 h, dehydrated with graded acetone series, and stained with saturated uranyl acetate. After dehydration with graded acetone series again and embedding in Epon, ultrathin sections (60–70 nm) were cut using an ultramicrotome (Leica UC7, Leica) and observed under a transmission electron microscope. The endfeet area of astrocytes and the average width of the endothelial tight junction were analyzed using the ImageJ software. The width of each tight connection is calculated at 5 points of equal proportion, and its average value is the average width of the tight connection.

### Acquisition of public gene sets

The gene sets for cerebrospinal fluid circulation and mitochondrial respirasome were obtained from GO:0090660 and GO:0005746, respectively (GO; Gene Ontology, geneontology.org).

### RT-qPCR

Total RNA was extracted from the hypothalamus tissues using Molpure^®^ TissueTotal RNA Kit (YEASEN Biotechnology (Shanghai) Co., Ltd.). In addition, the cDNA was synthesized using a gDNA digester plus (YEASEN Biotech Co., Ltd.). Then, the Hieff UNICON^®^ Universal Blue SYBR Green Master Mix (YEASEN Biotech Co., Ltd.) was used to execute an amplification reaction in Gene-9660 System (Bioer Technology, Hangzhou, China). The amplification conditions were 95°C for 5 min, 40 cycles at 95°C for 10 s, and 60°C for 30 s. Relative fold change was calculated using the −2ΔΔCT method using *Gapdh* as the control marker. Primer sequences are demonstrated in Supplementary Table 1.

### Statistical analysis

Data are presented as the mean ± SD. Normality was tested using the Shapiro-Wilk test. The behavioral data were analyzed by one-way repeated measures ANOVA. A one-way ANOVA and the least-significant difference (LSD) were used to determine differences between groups. All data analysis was conducted using the SPSS 26.0 software (SPSS Inc., Chicago, USA) and GraphPad Prism 8 (GraphPad Prism Software Inc., San Diego, USA). A *P*-value of < 0.05 was considered statistically significant.

## Results

### The water maze score and ST characteristic distribution in the moxibustion group were closer to those of mice in the WT group

In the MWM test, there was almost no difference between the APP/PS1 + MOX group and the APP/PS1 group on the first day (Figure 1C). However, on the second day, the time for mice in the APP/PS1 + MOX group to find the platform was shortened by 1/3, which was significantly different from that of mice in the APP/PS1 group (Figure 1C). In the stage of the hidden platform, although the time for the three groups of mice to find the platform continued to reduce, the training effect of mice in the APP/PS1 + MOX group was significantly better than that of the APP/PS1 group (Figure 1D). In the final stage of platform removal, the stay time in the target quadrant and the number of times crossing the platform in the APP/PS1 + MOX group were significantly better than those in the APP/PS1 group (Figures 1E, F). This showed that the learning and memory abilities of mice in the APP/PS1 group have been improved after moxibustion.

To roughly explain the gene change in different groups, we analyzed the ST gene profile of the whole mouse brain in each group. First, UMAP cluster analysis showed that the WT group and the APP/PS1 group can be significantly separated, indicating that the gene expression patterns of most cells are significantly different (Figure 1G). After graph-based clustering, 10 cell clusters were obtained (Figure 1H). According to the location information of each cell cluster in the ST and mouse brain atlas (Franklin and Paxinos, 2007), we matched these 10 cell clusters to 10 brain regions, including the cortex, the hippocampus, the thalamus, the white matter, the choroid plexus, and the ependyma.

### *Aqp4* in perivascular astrocyte and BBB reduced significantly in APP/PS1 mice, which was more obvious than that of *Gfap*

To further analyze the changes in *Aqp4* in perivascular astrocytes and BBB in each group, we selected *Gfap* and *Aqp4* to label astrocytes and selected *Pecam1* and *Flt1* to label BBB according to literature and the spatial cell adjacency (Figure 2A). The results showed that the expression of *Aqp4* in perivascular astrocytes of the APP/PS1 group decreased (*P* < 0.01), and this trend also existed in the BBB (Figure 2B). The number and proportion of astrocytes with high expression of *Aqp4* around vessels in the APP/PS1 group also decreased (Figure 2C). In addition, the brain regions of the cortex, the hippocampus, and the hypothalamus decreased significantly (*P* < 0.05, Figure 2D).

There are visible morphological changes in astrocytes in the brains of patients and mice with Alzheimer’s disease (Staurenghi et al., 2021). To further clarify the relationship between AQP4 and astrocytes, we calculated the correlation (or fitting rate) between AQP4 and GFAP, which is an intermediate filament protein integral to cytoskeletal dynamics. We found that the slope of the fitting line in the APP/PS1 group was lower than that in the WT group, and the correlation between *Aqp4* and *Gfap* was also reduced (Figure 2E, Pearson analysis, WT group: *R* = 0.575, *P* < 1 × 10^–39^; APP/PS1 group: *R* = 0.363, *P* < 1 × 10^–8^; merge: *R* = 0.55, *P* < 1 × 10^–99^). It showed that the expression of *Aqp4* in perivascular astrocytes of APP/PS1 mice was still decreased under the same morphological changes. In addition, in APP/PS1 mice, the relationship between *Aqp4* and morphological changes became more unstable.

We then analyzed AD mouse ST data of various ages (Chen et al., 2020) and single-cell RNA sequence data coming from patients with AD (Yang et al., 2022). In normal mice, the expression of *Aqp4* in perivascular astrocytes of 6-month-old mice was the highest. In contrast, it was significantly reduced in 6-month-old AD model mice (Figure 2F). In human hippocampal vessels, the expression of *Aqp4* in astrocytes of patients with AD was also significantly lower than that of normal elderly people (Figure 2G, *P* = 1.4 × 10^–16^), suggesting that the expression of *Aqp4* usually increases with the prolongation of the astrocyte process, but this increasing trend is weakened in AD.

### The ratio of *Aqp4* reduced in APP/PS1 mouse BBB endfeet, especially in the hypothalamus

**FIGURE 3:**
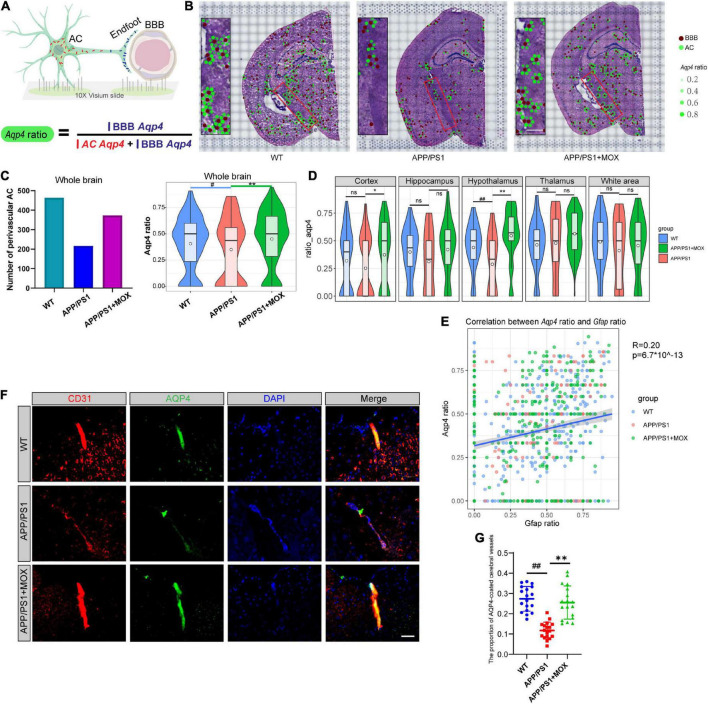
The reduction of the *Aqp4* ratio in APP/PS1 mice. **(A)** Schematic diagram of the calculation method of *Aqp4* ratio, which represents the *Aqp4* mRNA transport capacity of astrocyte to endfoot. **(B)** Brain maps of the *Aqp4* ratio. The brown points represent BBBs, and the green points represent ACs. The size and alpha of the green points represent the magnitude of the *Aqp4* ratio. Scale bar, 0.3 mm. **(C)** Barplot of the number of perivascular AC and violin plots of *Aqp4* ratio in brains. **(D)** Violin plots of *Aqp4* ratio of different brain regions. **(E)** A scatter diagram showing the correlation between the *Aqp4* ratio and *Gfap* ratio in AC. R, Pearson’s correlation coefficient; p, Pearson’s correlation test *p*-value. **(F)** IF analysis using CD31 and AQP4 antibodies of the brain. Blue, DAPI; red, CD31; green, AQP4. Scale bar, 40 μm. **(G)** Dot plot of the proportion of AQP4-coated cerebral vessels (*N* = 18). One-way analysis of variance with post-test ^#^*P* < 0.05, ^##^*P* < 0.01, APP/PS1 vs. WT; *P < 0.05, **P < 0.01, APP/PS1 + MOX vs. APP/PS1.

Considering that *Aqp4* mRNA needs to be transported to the terminal endfoot for assembly and late protein translation (Boulay et al., 2017), we further analyzed whether the reduced *Apq4* mRNA was more obvious in the endfeet of BBB, which may be evidence of the polar distribution of *Aqp4* mRNA in astrocytes and dysfunction of *Aqp4* mRNA transport to the endfeet of BBB in AD. Here, we introduced a calculation formula for the *Aqp4* ratio, which is determined as the proportion of *Aqp4*/BBB in the sum of *Aqp4*/BBB and *Aqp4*/AC (Figure 3A). The results showed that the number of astrocytes expressing *Aqp4* near the BBB in the APP/PS1 group was significantly less than that in the WT group, and the *Aqp4 ratio* in the APP/PS1 group was significantly reduced (Figures 3B, C Fold change = 0.825, *P* < 0.05). Moreover, in the hypothalamus region of APP/PS1 mice, the *Aqp4* ratio decreased most significantly (Fold change = 0.66, *P* = 0.006). To eliminate the effect of terminal deletion of astrocytes on this ratio, we conducted a correlation analysis of the *Aqp4* ratio and *Gfap* ratio (the proportion of *Gfap*/BBB in the sum of *Gfap*/BBB and *Gfap*/AC). It was found that the *Aqp4* ratio is almost independent of the *Gfap* ratio (Figure 3E, Pearson analysis: *R* = 0.20, *P* = 6.7 × 10^–143^). This result suggested that the change in the *Aqp4* ratio in APP/PS1 is not caused by the loss of the astrocyte terminal.

To verify this change in AQP4 around cerebral vessels at the protein level, we carried out co-labeled IF of CD31 and AQP4. It was found that the proportion of AQP4-coated cerebral vessels in the APP/PS1 group was significantly lower than that in the WT group (Figures 3F, G *P* < 0.01), indicating that the *Aqp4* mRNA transport capacity decline has affected the translation of AQP4.

**FIGURE 4:**
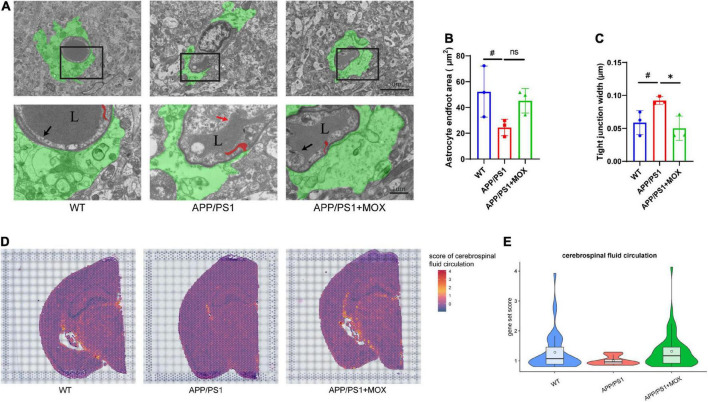
The reduction of the astrocyte endfoot area and cerebrospinal fluid circulation level in APP/PS1 mice. **(A)** Transmission electron microscopy images of the BBB. Green areas indicate astrocyte endfoot; red areas indicate endothelial tight junctions; black arrows point to normal endothelial lining; red arrow point to damaged endothelial lining; L, vascular lumen. Scale bar, 5 μm and 1 μm. **(B,C)** Barplot of astrocyte endfoot area and tight junction width (*N* = 3). **(D)** Brain maps of cerebrospinal fluid circulation level (GO:0090660). **(E)** Violin plots of cerebrospinal fluid circulation level (gene set score > 0.8). A one-way analysis of variance with post-test ^#^*P* < 0.05, APP/PS1 vs. WT; *P < 0.05, APP/PS1 + MOX vs. APP/PS1.

Next, we used TEM to evaluate the endfeet area of astrocytes around the cerebral vessels of mice and the structural changes to the BBB in AD. The endfeet area of astrocytes and the degree of blood vessels wrapped by the endfeet in APP/PS1 mice decreased significantly (Figures 4A, B). In endothelial cells, the endothelial lining of APP/PS1 mice was broken and discontinuous, which was more blurred than that in the WT mice. In addition, the tight junction between endothelial cells of APP/PS1 mice became loose and irregular, and their average width increased significantly (Figure 4C). The loss of perivascular AQP4 in the aging mouse brain resulted in impairment of perivascular CSF recirculation and Aβ clearance (Kress et al., 2014). A gene set (GO:0090660) was used to score in ST to explore the effect of reduced *Aqp4* polarization on cerebrospinal fluid circulation. The results showed that APP/PS1 mice showed lower levels of cerebrospinal fluid circulation not only in the periventricular area but also in other areas (Figures 4D, E).

**FIGURE 5:**
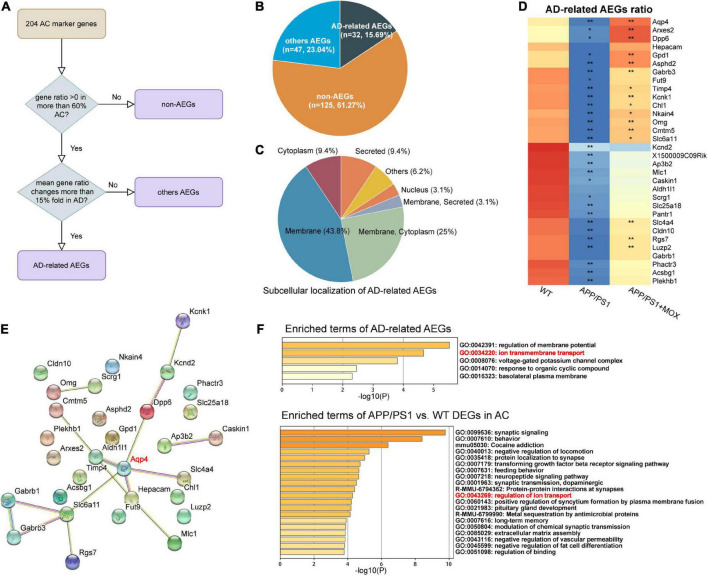
Other astrocyte endfoot genes changed in perivascular AC. **(A)** Flowchart of astrocyte endfoot gene (AEG) screening. **(B)** Pie chart indicating the proportion of AD-related AEGs to total AC marker genes. **(C)** Classification of AD-related AEGs based on subcellular localization analysis. **(D)** Heatmap of AD-related AEG ratio. All AD-related AEG ratios have a larger reduction in the APP/PS1 group. One-way analysis of variance with post-test *P < 0.05, **P < 0.01 APP/PS1 vs. WT and APP/PS1 + MOX vs. APP/PS1. **(E)** Protein–protein interaction (PPI) network constructed of AD-related AEGs using STRING. Among them, *Aqp4* is located in the central position. **(F)** Pathway enrichment analysis of AD-related AEGs and AC-DEGs. Both of them enriched the pathway of ion transmembrane transport (GO:0043269).

**FIGURE 6:**
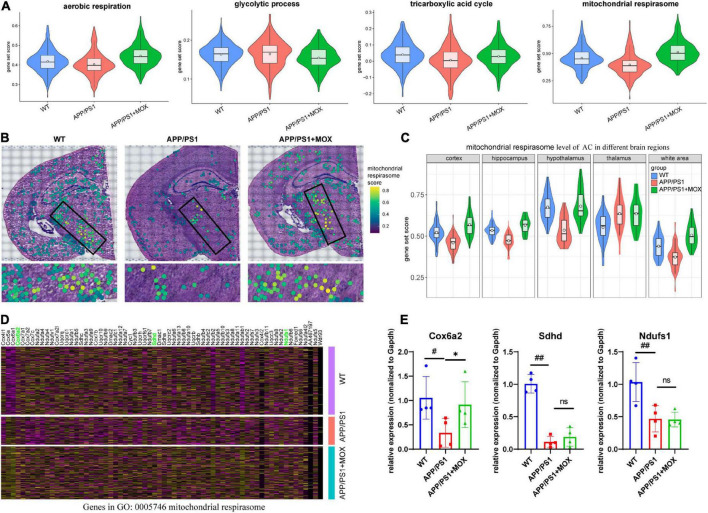
The change in the mitochondrial respirasome level in perivascular astrocytes. **(A)** Violin plots of energy-related gene set score. The most significant change was observed in the mitochondrial respirasome. **(B)** Brain maps of mitochondrial respirasome levels. **(C)** Violin plots of mitochondrial respirasome levels in different brain regions. **(D)** A heatmap showing the DEG expressions, which were enriched for the pathway of mitochondrial respirasome (GO:0005746). **(E)** RT-qPCR analysis of mitochondrial respiratory chain enzyme mRNA *Cox6a2*, *Sdhd*, and *Ndufs1* (*N* = 4). One-way analysis of variance with post-test ^#^*P* < 0.05, ^##^*P* < 0.01, APP/PS1 vs. WT; *P < 0.05, APP/PS1 + MOX vs. APP/PS1.

The above results show that *Aqp4* mRNA may have a transport disturbance from the cell body to the BBB endfoot. Does this phenomenon only occur in *Aqp4*? What is the potential mechanism? To explore the answers, we next used the spatial transcriptomic data to evaluate the polarity distribution of other mRNAs in BBB and analyzed the change in energy metabolism genes in different brain regions. First, we selected 204 astrocyte cell markers of BBB and obtained 79 AEGs with a gene ratio greater than 0 in more than 60% of the AC. Among them, 32 AD-related AEGs were screened out with an average gene ratio change greater than 0.15-fold in the APP/PS1 group (Figures 5A, B and Supplementary Table 2). In addition, 71.9% of AD-related proteins of AEGs were distributed in the membrane, which provided strong evidence for the high correlation between AEGs and astrocyte endfoot (Figure 5C and Supplementary Table 3). Other AD-related AEG ratios showed a low expression in the APP/PS1 group (Figure 5D). Among them, *Aqp4*, *Gabrb1*, *Gabrb3*, *Kcnd2*, *Kcnk1*, *Slc4a4*, and *Slc6a11* are inorganic molecular entity transmembrane transporters; *Dpp6*, *Rgs7*, and *Slc25a18* can regulate membrane potential; and *Cldn10* and *Nkain4* are voltage-gated potassium channel complex genes. In the protein–protein interaction (PPI) network, *Aqp4* was the core gene in these AD-related AEGs, which enhanced the representativeness of *Aqp4* (Figure 5E). Notably, the ion transport-related term was enriched not only from AD-related AEGs but also from DEGs of perivascular astrocytes in the APP/PS1 group (Figure 5F and Supplementary Tables 4, 5). This indicated that there were more ion transport-related genes had expression changes in the whole astrocyte. We then used the aerobic respiration gene set to score perivascular astrocytes and found that there was no significant change in the APP/PS1 group during aerobic respiration (Figure 6A). However, in the aerobic respiration splitting process, we found that the mitochondrial respiratory chain genes decreased significantly in the APP/PS1 group (Figure 6A). Similar to the change in the *Aqp4* ratio, the hypothalamus was the most obviously changing region of the mitochondrial respiratory chain in the APP/PS1 group (Figures 6B, C). Then, we analyzed the mRNA changes in ST data of COX6A2, SDHD, and NDUFS1, major enzymes in the mitochondrial respiratory chain, and carried out RT-qPCR validation. The results showed that genes of COX6A2, SDHD, and NDUFS1 in the APP/PS1 group were expressed significantly low (Figures 6D, E and Supplementary Table 6).

### Moxibustion can partially restore the abnormal polarization distribution of *Aqp4* in BBB endfeet

Compared with the APP/PS1 group, the cell expression pattern of the APP/PS1 + MOX group is more similar to that of the WT group (Figure 2A). In the study of perivascular astrocytes, it was found that moxibustion can increase the expression of *Aqp4* (Figures 2D–G) and increase the *Aqp4* ratio, especially in the cortex, hypothalamus, and white matter (Figures 3B–D). After moxibustion, the slope of the fitting line of perivascular astrocytes in APP/PS1 mice and the correlation between *Aqp4* and *Gfap* also decreased (Figure 2G, Pearson analysis, APP/PS1 group: *R* = 0.373, *P* < 1 × 10^–7^; APP/PS1 + MOX group: *R* = 0.618, *P* < 1 × 10^–37^). Subsequent IF and TEM experiments also showed that moxibustion could improve the decrease in AQP4 around the blood vessels of APP/PS1 mice (Figures 3F, G, *P* < 0.01), increase the endfoot area of astrocytes, reduce the tight connection width between vascular endothelial cells, and improve the level of cerebrospinal fluid circulation (Figures 4A–E). In addition, our data also showed that moxibustion can effectively increase the polarization of most AD-related AEGs, which are closely related to *Aqp4* (Figure 5D). Moreover, moxibustion can improve the low expression of mitochondrial respiratory chain genes in APP/PS1 mice and effectively increase the expression of Cox6a2 in the hypothalamus (Figures 6A, D, E).

## Discussion

This study found that moxibustion can increase the ratio of *Aqp4* in BBB astrocyte endfeet in the hypothalamus region, increase the expression of key enzymes in the hypothalamic mitochondrial respiratory chain, and also improve the abnormal distribution of other mRNA related to transport. Besides, we found that genes related to the circulation of cerebrospinal fluid increased.

We first used ST to screen the BBB, composed of various endothelial cells, pericytes, astrocyte endfeet, etc. (Banerjee et al., 2016). Then, according to the feature that the distance between the two spots in the 10X space transcriptome chip is 45 microns and the length of astrocyte processes can reach 90 microns (Lange et al., 2018) (that is, the rationality of the distance), the astrocyte bodies adjacent to the BBB, which include astrocyte endfeet, were located. This method partly solved the problem that it is difficult to isolate astrocyte bodies and endfeet related to BBB anatomically by traditional methods and facilitated the analysis of the change of astrocyte endfeet in mRNA level. Second, we used the *Aqp4* ratio and fitting rate, two indicators that could indirectly reflect the polar distribution of *Aqp4* in astrocytes. Besides, we technically avoided the influence of astrocytes in the same spot or repeated calculation of astrocyte bodies in the BBB spot, which may be affected by the morphological changes of astrocytes in AD. Therefore, the distribution of *Aqp4* in BBB other than in the whole astrocytes was accurately calculated. As we speculated, the results showed that the fitting line slope of *Aqp4* and *Gfap* expression in the APP/PS1 group was significantly lower than that in the WT group and the APP/PS1 + MOX group (Figure 2E), indicating that the low *Aqp4* expression in astrocyte body did not relate to the overall changes in astrocytes in APP/PS1 mice. In addition, further analysis of the correlation between the *Aqp4* ratio and *Gfap* ratio showed that the correlation was small (merge: *R* = 0.2), and the slope of the fitting line was smaller than that of *Aqp4* and *Gfap* (Figure 3E). These results demonstrated that the change in the *Aqp4* ratio was mainly influenced in the endfoot of BBB because of the overall astrocytes with a relatively constant expression of *Gfap* but an unstable expression of *Aqp4*. From the above results, we can infer a polar distribution of *Aqp4* mRNA in the BBB of APP/PS1 mice. Then, we analyzed the data of AD mice and patients with cognitive impairment in the public database and found that our data were consistent with the results of the public database analysis, which verified our hypothesis that the polarization distribution of *Aqp4* is obvious in AD. Moreover, in the APP/PS1 + MOX group, we found moxibustion can improve the polar distribution of *Aqp4* mRNA in perivascular astrocytes.

The current study showed that moxibustion can improve the polarization of AQP4 and reduce the impairment of learning and memory abilities in APP/PS1, which provides new evidence that moxibustion can play multiple roles in treating AD. Previous clinical and experimental studies showed that many mechanisms of acupuncture and moxibustion are involved in delaying the occurrence and development of AD. For instance, they can reduce APP and the deposition of SPs in the brain (Yang et al., 2019), enhance glucose metabolism in AD mice hippocampus (Cao et al., 2017), inhibit synaptic degeneration and neuroinflammation (Cai et al., 2019), promote nerve regeneration and synaptogenesis (Zhao et al., 2020), inhibit neuronal apoptosis, and promote a neuroprotective effect (Tang et al., 2020). Our previous studies also proved that moxibustion can reduce the levels of Aβ, tau, and p-tau proteins both in the hippocampal and cortical regions (Huang et al., 2022), promote the astrocyte-neuron interaction, and enhance synaptic plasticity of APP/PS1 mice (Zhang et al., 2022). However, this is the first time that we have found that moxibustion can influence the polarization of AQP4 and other molecules.

**FIGURE 7:**
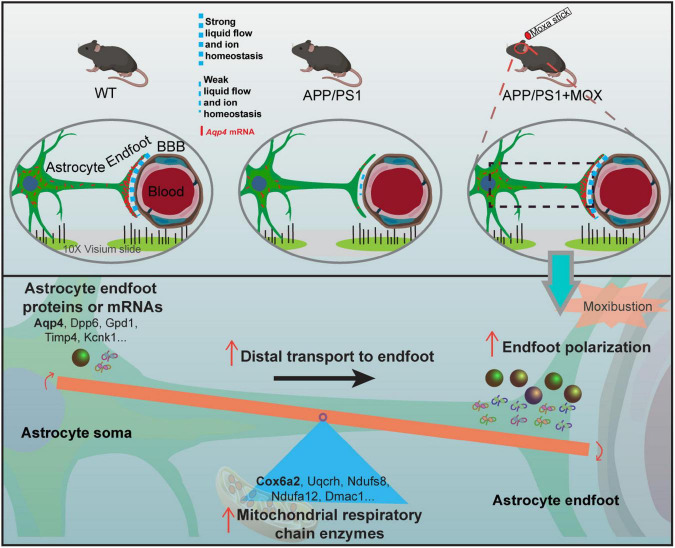
The mechanism diagram of moxibustion improving *Aqp4* polarization around cerebral vessels in APP/PS1 mice.

We also found that the hypothalamus was the main brain region with the polar distribution of *Aqp4* mRNA. Since the hypothalamus is an important brain region where the energy metabolism disorder occurs, we analyzed the genes related to brain energy metabolism. The results showed that the expression of a large number of metabolic genes in ST data decreased, and the hypothalamus was the most obvious region too. Previous studies confirmed that the hypothalamus is involved in sleep abnormalities in patients with AD and mice (Musiek and Holtzman, 2016). Sleep abnormalities will hinder the removal of tau protein and amyloid-β precipitation (Kang et al., 2009; Osorio et al., 2016). Therefore, the hypothalamus may be a critical therapeutic target in AD. Hypothalamus is an important brain region for moxibustion to work. For instance, many studies showed that moxibustion can regulate hypothalamic rhythm activities and rhythm genes such as CLOCK and BMLA1 (He et al., 2022). Besides, the regulation of moxibustion on the hypothalamic HPA axis has also been extensively reported (Ma et al., 2016). Our previous studies also showed that moxibustion can relieve anxiety in colitis mice by reducing the secretion of corticotropin-releasing hormone in the hypothalamus (Wei et al., 2019). We infer that the effect of moxibustion on the disorder of energy metabolism in the hypothalamus in this study may lead to further enhancing transport proteins and mRNA to the endfeet of astrocytes (Figure 7). Our results seem to explain the result of a study by Chen et al. (2021), whose research showed that moxibustion could promote the cortical velocity of cerebrospinal fluid flow and the level of AQP4 in healthy mice.

This study also has some limitations. For example, one spot in the 10X space transcriptome chip has a diameter of 55 microns, and the diameter of capillaries in the brain is between 5 and 10 microns. Therefore, small cells such as vascular endothelial cells cannot be separated independently, which may affect the current results. Therefore, more studies are needed to verify our findings and inferences in the future.

To sum up, we used ST to study the distribution of *Aqp4* in the BBB of APP/PS1 mice before and after moxibustion. According to the ratio of gene expression in the endfeet of the BBB and the cell bodies of astrocytes, we found 32 AD-related AEGs with *Aqp4* as the core. It was found that there was a polar distribution loss of the *Aqp4* gene in astrocytes endfeet in APP/PS1 mice. Disorder of the mitochondrial respiratory chain may be the reason for this abnormal distribution, and the above results are most obvious in the hypothalamus. Moxibustion can improve the mitochondrial respiratory chain disorder of APP/PS1 mice, and the polarity distribution of *Aqp4* is also significantly improved. These results suggest that hypothalamic *Aqp4*-related therapy may be an important target to improve cognitive impairment.

## Data availability statement

The data presented in this study are deposited in the NCBI’s Gene Expression Omnibus repository, accession number: GSE222981 (https://www.ncbi.nlm.nih.gov/geo/query/acc.cgi?acc=GSE222981).

## Ethics statement

This animal study was reviewed and approved by the Animal Care and Use Committee of Chengdu University of Traditional Chinese Medicine.

## Author contributions

SL, HL, QW, and LX drafted the study. QW conceived and designed the experiments. SL, HL, YS, JW, NZ, CL, and LX performed the experiments. SL, WZ, YW, and QW analyzed the images and data. All authors read and approved the final manuscript.

## Footnotes

1
http://www.ncbi.nlm.nih.gov/geo/
